# Statistical Extraction Method for Revealing Key Factors from Posture before Initiating Successful Throwing Technique in Judo

**DOI:** 10.3390/s21175884

**Published:** 2021-09-01

**Authors:** Satoshi Kato, Shinichi Yamagiwa

**Affiliations:** 1 Doctoral Program in Computer Science, University of Tsukuba, 1-1-1 Tennodai, Tsukuba, Ibaraki 305-8573, Japan; kato@padc.cs.tsukuba.ac.jp; 2 Faculty of Engineering, Information and Systems, University of Tsukuba, 1-1-1 Tennodai, Tsukuba, Ibaraki 305-8573, Japan; 3 JST, PRESTO, 4-1-8 Honcho, Kawaguchi, Saitama 332-0012, Japan

**Keywords:** motion sensing, skill extraction, throwing technique of judo, statistical approach, coaching technique

## Abstract

Many methods such as biomechanics and coaching have been proposed to help people learn a certain movement. There have been proposals for methods to discover characteristics of movement based on information obtained from videos and sensors. Especially in sports, it is expected that these methods can provide hints to improve movement skills. However, conventional methods focus on individual movements, and do not consider cases where external factors influence the movement, such as combat sports. In this paper, we propose a novel method called the Extraction for Successful Movement method (XSM method). Applying the method, this paper focuses on throwing techniques in judo to discover key factors that induce successful throwing from the postures right before initiating the throwing techniques. We define candidate factors by observing the video scenes where the throwing techniques are successfully performed. The method demonstrates the significance of the key factors according to the predominance of factors by $\chi^{2}$ test and residual analysis. Applying the XSM method to the dataset obtained from the videos of the Judo World Championships, we demonstrate the validity of the method with discussing the key factors related to the successful throwing techniques.

## 1. Introduction

Sports are a fun activity which help us to maintain a healthy body. When we want to be a good player in a sports activity, we put a lot of effort into acquiring key movements of specific motor skills. Based on the advice of experts, a good method to become a good player is to learn the key movements by repetition. However, this approach requires a long time to acquire the skills unless we know the key movements. Therefore, it can reduce motivation to play the sport itself. Thus, we need some methods that motivate us to put the effort in for hard training.

Coaching [1] and training [2] theories help us to acquire the key movements for the target motor skills in a shorter training time. The theories attempt to build concrete systems regarding effective training for motor skills. Athlete-centered exercise [3] is one of the recent trends in the field. We can learn the effective motor skills depending on the game situations in team sports. However, those theories indispensably require skillful trainers or coaches. They also need to be trained for teaching practical skills that depend on player characteristics. For example, the teaching methods are focused on improving skills for the entire team rather than for individuals. By selecting the appropriate methods from experimental examples such as [4,5], the trainers and the coaches have to find problems. Furthermore, they need to lead a team to succeed in the target movements with minimal training. We can apply the same strategy to training skills for individuals. For example, it is necessary for the trainer and the coach to understand the differences among athletes’ physical abilities and to be familiar with how to convey them the know-how for the motor skills by selecting suitable training methods. However, it is difficult to define concrete methods that convey the key factors for the target movements to athletes. Therefore, the methods depend on experience and philosophy of the trainers or coaches. It is also difficult to find a method that matches the athlete.

According to the conventional training methods discussed above, if we already knew obvious key points of the target movement, we are able to improve the skill. Additionally, we have a possibility to develop an automated system that serves coaching and training guides based on such key points. Recently, watching performances of advanced athletes from videos has been considered as one of valid methods. We can understand the key points from the performances by watching videos repeatedly and can try to apply them to our own movements [6]. However, this method inevitably requires specialized knowledge of the target movements and relies on advice associated with experiences of advanced athletes. This also depends on personal interactions. To eliminate the interaction, methods to measure skills objectively by using inertial sensors have been proposed such as [7]. The method statistically compares between performances of advanced athletes and novices, and defines equations for the models for comparing numerically the target movements. We can also find a method with a machine learning approach [8]. It exploits key points of a target movement between advanced athletes and novices. Thus, we consider these methods as effective in mobile and IoT devices available in the market because the methods provide fundamental technologies for automatic coaching and training methods.

However, there exists a problem that these methods are not applicable to combat sports such as judo, wrestling, boxing etc. These combat sports include interactions between players. Therefore, when an event such as scoring one occurs after a movement of one player with some influences from the opposed player, those methods are not able to derive the obvious key factor for the event. Therefore, we focus on a technique that derives the key factors in combat sports. We expect that the technique discloses the key factors for scoring events. The technique will give us opportunities to analyze weaknesses of the opposing players and develop new knowledge in coaching theory. Moreover, if the analysis using videos becomes available, coaching will be automated in combat sports.

This paper proposes a novel method that derives key factors for effective learning of a movement in combat sports. We will focus on judo as a high-impact example of combat sports and also propose a method to derive the key factors for successful throwing techniques. The throwing technique is an important motor skill as a scoring event for winning in judo. The players want to master the skill fluently. In this paper, we will focus on postures before successful throwing techniques. Then, we propose a statistical method. The method will derive the key factors that induce successful throwing techniques from the posture right before the throwing techniques.

Contributions and new findings of this research are listed as follows.

- We have developed a system based on a statistical approach, called the Extraction for Successful Movements method (XSM method), that extracts the key factors by applying a dataset of available factors from body parts at a posture before a target movement. The dataset is captured from throwing scenes in videos recorded during matches.
- We have developed a method that finds differences among groups by projecting a subset of the dataset against attributes (gender difference, weight class, etc.). This function is included in the XSM method.
- According to effective results experimentally demonstrated regarding the throwing technique in judo, we have proved that the XSM method is able to becomes an effective method that finds key factors for a successful target movement.

This paper is organized as follows. In the next section, we will describe backgrounds and definitions of this research. In Section 3, we will propose the novel method for finding key factors that induce successful movements from posture before an initiating successful movement. In Section 4, we will perform experimental evaluations for the method using a dataset from judo. Finally, we will conclude this paper.

## 2. Backgrounds and Definitions

### 2.1. Methodologies for Learning Motor Skills in Sports

When a person is going to learn a certain movement, observing an exemplary performance of the movement by another person is helpful. Miner has insisted that learning of motor skill begins first through sight, and also, the most effective method is to show a successful movement to the learner during the learning process [9]. Indeed, recent modern training is based on observing target movements from videos. The trend is considered as one of the most important training methods to let the learners understand their own movements [6,10,11,12,13]. For example, [6] proposes that rapid feedback of video recorded during a match is important for providing advice from a coach to players right after the game. Ref. [10] introduces a coaching method that applies to show video to some case studies. Ref. [11] insists that the video is applicable to physical education. In [12], it is experimentally proposed that the training method for golf swings based on videos is the most effective one, while it needs a long time, by comparing three methods—a video-based instruction, a verbal one and a self-guided one. Besides these positive effects of video-based training, the movements can have various patterns depending on a player’s physical characteristics and their condition. Therefore, it is necessary to observe the movements from multiple scenes of various videos and find common key factors. However, it is not able to define an absolute method to find the factors due to objective observation by coaches and trainers. Thus, we need a method that eliminates the biased interpretation of the coach and also, develop a system that generates absolute advice. Regarding the objective observations, Ref. [13] reports that it is not always effective to use videos as a learning method for novices of tennis service. This result obviously shows that the factors focused on novices do not become the key ones. Particularly, it is impossible to define the absolute focusing points on sports such as figure skating, synchronized swimming, ski mogul, etc. which evaluate beauty, dynamism and elegance. Therefore, it is necessary to develop a method that extracts the key factors of a target movement from videos based on an objective observation and provides feedback to the learners effectively.

In order to objectively acquire key points of target movements from video, several novel methods have been proposed for acquiring coordinates of the points based on physically measured data from sensor devices or optical sensors. The researches [14,15] are the typical trials for measuring the body movements numerically. Furthermore, there are advanced researches that apply measure sports activities via sensors. For example, in [16], focusing on badminton smash, the authors derive differences of the strengths between genders from three-dimensional coordinate values of the arm movements captured by camera. They insist that the higher racquet grip velocity induces a more rapid smash. Furthermore, they posit that rapid smashes in the case of male players are caused by the movements of the shoulder abduction and elbow extension. In [17], the authors use motion sensors to capture objective data from the golf swing. They derive the key points of the motion by the sensors attached to totally five points on a golf club and the participant’s body. They propose a quantitative model function to find key factors among the local features of the sensor locations with applying PCA (principal component analysis) and LDA (Local Discriminant Analysis) [18]. In [19], the authors analyze the center of gravity between both feet during a golf swing using two force plates. Furthermore, they derive the importance of the center of gravity regarding the front foot. In [7], the authors propose a method that automatically determines skills of skiing and snowboarding by applying motion sensors in smartphone. They focus on factors including tempo, symmetry, and dynamism that cause visual elegance of the movements. The users of the system can track their skills numerically by using the accelerometer in the smartphone and evaluate their own skills from the result according to the mathematical models. During the research, the authors have set maximal values from the statistical values acquired from advanced players. However, it causes another problem to be addressed. We are not able to find absolute maximums from a small dataset due to the large diversity of human motor skills. Therefore, in [8], the authors define the distance of skills between novices and experts as the differences of movement data patterns. They apply a machine learning approach that drives the *skill distances* of the data patterns captured from movements. We can apply a set of data in a timeline as the captured data for the skill distances such as two-dimensional vectors plotted from video frames and linear data from motion sensors. According to the skill distances, we can make groups of the dataset with similar skills. It derives the key factors related to measurement points. Thus, the method lets us find the key factors that distinguish between beginners and advanced players.

As described above, although we confirmed that the training methods with video provide effective outcomes, there exists the case with a negative effect when subjective opinions of coaches and trainers are included in the analysis. In order to eliminate the subjective opinions, there are attempts to quantify the skills from the model formulas using the coordinate values from video frames and the measured data from the sensor devices. Those methods derive the key factors of the target movements by using mathematical and/or machine learning approaches. Now, it has become successful to accurately analyze the movement from the video based on such objective information. However, we are able to apply those methods to derive only the key factors of individual’s motor skills. Thus, we are not able to apply the methods directly to combat sports that are played by multiple persons influenced by each other. Because the learner is influenced by movements from the opponent player, it is hard to model the analyzing system. If we can address the problem, we will be able to establish a new coaching method that improves motor skills strongly related to scoring for winning and also that plans strategies before matches. Thus, it is important to develop a novel method with an objective approach that derives the key factors of movements in combat sports.

### 2.2. Classifications and Training Methods for Throwing Techniques in Judo

Here, let us focus on judo as a typical example of combat sports. Judo matches are performed with two players. The winner is decided by scores called *Waza-ari* and *Ippon* according to throwing techniques, grappling techniques and penalties of the opponent player. Scoring techniques are defined in the International Judo Federation [20] rule book (a table of technique names in Japanese and their translations in English are also available in its appendix.). To win in a judo match aggressively, the player needs to master attacks and defenses regarding especially throwing techniques and increase the successive possibility of the techniques during their training. In order to learn the throwing techniques, it is effective for the player to understand the key factors that lead to the successful movements by repeating observations of video recorded in match games or training. Then, the knowledge regarding the key factors should inform the athlete’s movements. Thus, researchers have investigated classifications of the throwing techniques to find the factors as follows.

The founder of judo, Jigoro Kano, has originally defined a traditional classification of the throwing techniques. He has classified those focusing on four perspectives: hand, hip, foot and sacrifice techniques [21]. The first three ones are related to the parts of the body used at the center of the throw. The last one is focused on how the thrower discards their own body in the throw (called *Tori*). Furthermore, Kano has classified these into 68 different throwing techniques and has given names to the techniques according to the perspectives of, for instance, Tori’s positions of the grip, how Tori’s legs are performed and how the body of the throwee (called *Uke*) is thrown. In a match, Kano also defined the winner as the one who performs the successful throwing. On the other hand, Dopico et al. pointed out that Kano’s classification from the four types of throwing techniques was based on anatomical aspects in spite of kinematic or tactical aspects. They classified the throwing techniques into nine major types based on five practical aspects: movement structure, sustentation base, space where Uke is thrown, direction of the dynamic leg and spatial zone of the dynamic leg. They assigned those 68 different throwing techniques to the types [22].

In judo coaching, the above classifications are considered as the criteria for learning throwing techniques. However, the criteria are not defining factors that induce a successful throw, but the elements that organize a throwing. Therefore, several objective analyses have been proposed for the successful throwing techniques based on watching videos of match games. For example, Mayo et al. proposed a model to find the factors for successful throwing techniques based on the classification of Dopico’s motor criteria [23]. They investigated the factors from the scoring scenes brought by direct- and counter-attacks. They focused on the lateral structure and the position of both feet during successful throwing. However, this method analyzes movements after initiating a throwing movement. In other words, they investigated the key factors that lead to successful throwing after Tori has already selected which throwing technique to perform. The analysis focuses on the duration between the start and the end of the throwing. Therefore, this method does not bring us obvious coaching hints right before the throwing movement.

Additionally, in judo, we can find video-based biomechanical analysis. For example, Ref. [24] compares the speed of leg sweep using a kinematic model of *Harai-goshi* among novices and experts. Ref. [25] compares *Seoi-nage* movements among elite athletes and college judo players from a kinematical approach. Ref. [26] compares the angular velocity of the hips, knees, and ankles among novices and experts regarding the movement of leg sweep during *O-soto-gari*. These have revealed the key factors that lead to a successful throwing after the start of the throwing movement. Therefore, we are still not able to find a concrete posture before initiating a successful throwing technique. This also does not bring us an effective coaching method focusing on a situation before Tori tries to begin throwing Uke.

From the other aspect [27,28] propose systems that record the numbers and types of throwing techniques, as well as the time durations of *Kumite*, rest and throwing in entire matches by analyzing judo match videos. However, these systems do not clearly derive the key factors for initiating the throwing techniques. Thus, we never have sufficient knowledge for a systematic coaching method for successful throwing techniques.

### 2.3. Discussion

As described above, we confirmed that it is important to watch videos during training in sports. We also explained that it is important to identify key factors of movements according to objective information. To provide the objective information, we can find advanced methods by using sensing devices. However, it is hard to apply them to combat sports because the conventional methods were athlete-centered based on the analysis for individual athletes. Additionally, focusing on judo, the advanced research analyzed throwing techniques by categorizing those based on Tori’s and Uke’s postures during the throws. While learning the throwing techniques, Tori needs to master an ability to select one of the most successful throwing techniques dynamically in a match. However, the categorizations do not tell Tori the key techniques for selecting the best throw. The decision for selecting the throw must be done by both Tori’s and Uke’s postures right before initiating the throwing technique. Here, if we can know the key factors that induce the successful throwing technique, training by watching videos becomes more effective. Thus, we can develop a new training method for Tori to master the most successful throwing techniques if we find correlations among the postures right before initiating a throwing technique and possibilities for the success.

This paper will disseminate a novel method based on a statistical analysis method that derives mechanisms for scoring opportunities in combat sports. Focusing on the throwing techniques of judo, we will propose a method to extract factors that lead to successful throwing techniques from the postures of Tori and Uke right before initiating the throwing. We expect that the probability for the most successful throwing technique will vary according to combinations of various candidate factors caused by, for instance, hands and legs right before initiating the throwing. Our method will become an effective training tool in combat sports because we can just concentrate to learn the key factors with decreasing the number of body parts to be focused during watching videos.

## 3. Extraction for Successful Movement Method Applying to Judo

### 3.1. Overview of Method

**Figure 1:**
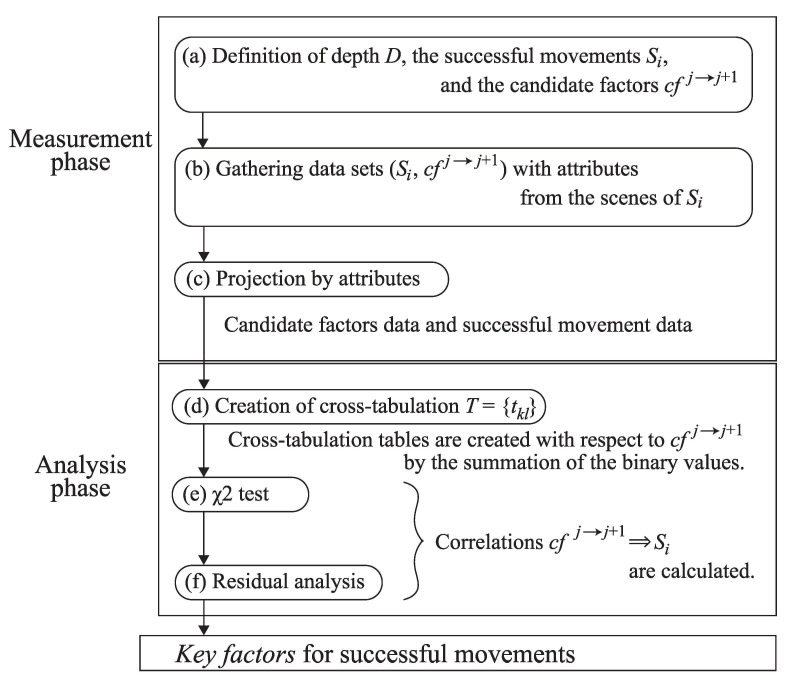
Phases and steps of the XSM method.

Figure 1 shows the steps of the method proposed in this paper, called the Extraction for Successful Movement method (XSM method). This method has two main phases, *measurement phase* and *analysis phase*. In the former phase, we gather the input data into the latter phase observed from the videos in which the target movements are successfully performed. According to the scenes, we obtain a dataset that contains the types of *successful movements*, *candidate factors* and *attributes*. The first ones are selected from the types of target movements to be analyzed. The second ones are selected from the potential candidate factors that can be the key factors before initiating the corresponding successful movements. The factors are defined by the combinations of the body parts that affect forces between the players in the scenes. The last ones are the information related to the scenes such as gender, height, weight and game records of the players.

The second phase analyzes the correlations among the successful movements and the candidate factors. Before the analysis, the dataset is projected by the attribute information to specify the group under some conditions. Then, the analysis outputs the key factors from the candidate factors that have strong correlations to successful movements.

Regarding the throwing techniques in judo, we apply the categories of throwing techniques for the successful movements, and the body actions of Tori and Uke for the candidate factors. We observe the factors from the scenes before initiating the throwing techniques. The analysis outputs the correlations between the successful throws and the factors. This helps us to understand which actions of Tori and Uke induce successful throws. Thus, during the coaching and training of a certain throwing technique, we can find the actions that the players should pay attention to as the key factors before initiating the successful throwing technique. This learning method can be used for iterative learning with video. Additionally, we can use the method for an effective analysis in a match. For example, the players can know the key factors derived from the analysis to avoid the critical situation when a player becomes Uke before initiating the successful throwing technique due to the Tori’s offense.

**Figure 2:**
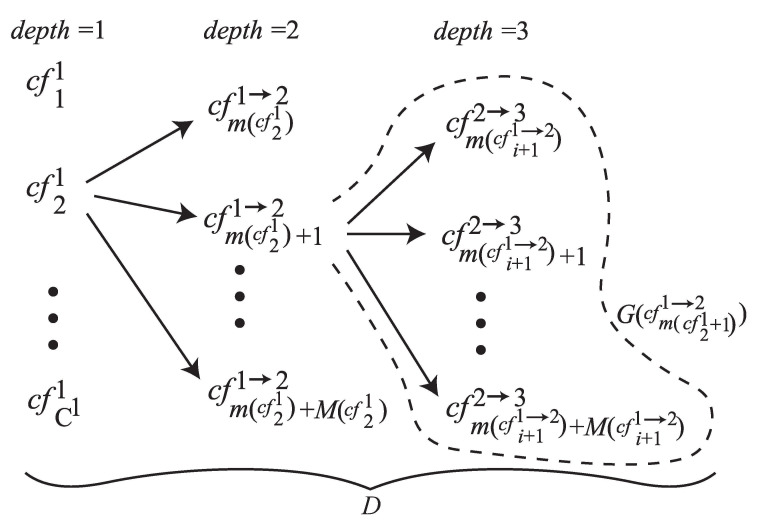
Example of the tree structure of the candidate factors.

Now, we explain the detailed flow of the XSM method. The flow is composed of six steps. The first step in Figure 1a determines the classification of the successful movements and the candidate factors. Let $S_{i}$ be the successful movements where 1≤i≤N. Regarding the candidate factors, we define the maximal depth D(≥1) for the granularity of the candidate factors. Incrementing the depth, we create a tree with the nodes from each candidate factor recursively as shown in Figure 2. In each depth *d*, we define the detailed candidate factors than the ones in d−1. This brings us the analysis for incremental candidate factors. Let d=1 by $cf_{j}^{1}$ be the *j*-th candidate factor at the first depth where $1\leq j\leq C^{1}$. The *j*-th candidate factor in the depth d=i+1 associated from the candidate factor in the depth d=i is denoted by $cf_{j}^{i\to i+1}$ where $1\leq j\leq C^{i\to i+1}$. The candidate factors from the same root $cf_{k}^{i-1\to i}$ is grouped in $G(cf_{k}^{i-1\to i})$. The number of the candidate factors included in the group is $M(cf_{k}^{i-1\to i})$. The first index of the candidate factors in the group is $m(cf_{k}^{i-1\to i})$. We also define the candidate factors in the depth d=D by $cf_{j}^{D}=\phi$ where $1\leq j\leq C^{D-1\to D}$ because there is no node from the factors. The analysis phase will derive the correlations $G\left(cf_{j}^{i\to i+1}\right)\Rightarrow S_{k}$ where $1\leq j\leq C^{i\to i+1}$ and 1≤k≤N. The next step shown in Figure 1b gathers combinations of $\left(S_{i},cf_{j}^{1}\right)$ in the depth d=1 where 1≤i≤N and $1\leq j\leq C^{1}$, and/or the ones of $\left(S_{i},cf_{k}^{j\to j+1}\right)$ in the depth 2≤d≤D where 1≤i≤N, 1≤j≤D and $1\leq k\leq C^{j\to j+1}$, with the attributes from the scenes of the successful movement. Each element of cf arrays has a binary value that stores if the candidate factor is occurred in the scene or not. The step (c) performs a projection by some attributes associated to the scenes. Assume that the number of total scenes is *K*, it is projected to $K^{\prime }$ ones where $K\geq K^{\prime }$. The total number of all combinations is calculated from the $K^{\prime }$ combinations of $S_{i}\times \left(C_{1}+\sum_{k=2}^{D}C^{k-1\to k}\right)$. The step (d) creates cross-tabulations $T\left(G\left(cf_{j}^{i\to i+1}\right)\right)=\left\{t_{kl}\right\}$ ($1\leq j\leq C^{i\to i+1}$, 1≤k≤N and $m\left(cf_{j}^{i\to i+1}\right)\leq l\leq m\left(cf_{j}^{i\to i+1}\right)+M\left(cf_{j}^{i\to i+1}\right)$) with respect to the candidate factors in depth d=i+1. The tabulation has the rows of the successful movements $S_{k}$ and the columns of the candidate factors $cf_{l}^{i\to i+1}$. The element $t_{kl}$ is a summation of the binary values of the corresponding candidate factor of the combinations gathered in the step (b) regarding ($S_{k}$, $cf_{l}^{i\to i+1}$). Here, the number of cross-tabulations is $C^{1}+\sum_{i=2}^{D}C^{i\to i+1}$. The step (e) performs $\chi^{2}$ tests against each cross-tabulation of the groups *G*. Finally, the step (f) performs residual analysis on the cross-tabulation containing the variables identified as the key factors. Thus, we obtain correlations between the classification of successful movements and the key factors.

As explained above, the XSM method is able to find the key factors systematically regarding the successful movements. To promote acquiring motor skills, we can apply the results to coaching and training techniques by observing the key factors of the learners. Thus, the XSM method will objectively determines the factors that cause the successful movements. Additionally, the method will provide a novel technique to acquire skills by focusing on the movements of the most important body parts by selecting those from the large diversity.

### 3.2. Selecting Candidate Factors for Successful Throwing Techniques in Judo

We define the types of throwing techniques as the successful movements and the candidate actions that lead to a successful throw as the candidate factors. Table 1 shows the candidate factors in each depth. We apply D=3.

**Figure 3:**
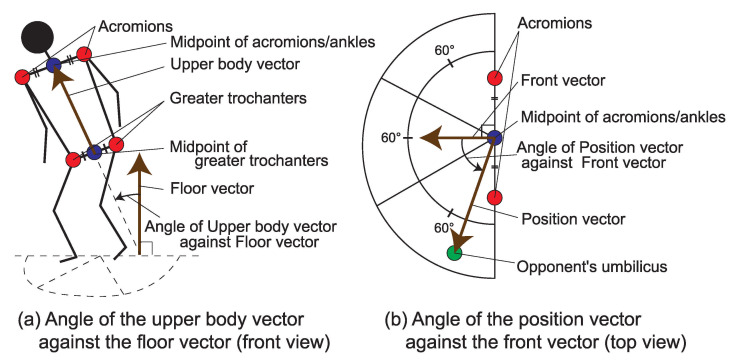
The angles and the vectors to determine the candidate factors. (**a**) The angle of the upper body vector against Floor vector from the front view. (**b**) The angle of the position vector against the front vector from the top view.

We use the four classifications of throwing techniques (hand, hip, foot and sacrifice techniques) defined by Jigoro Kano [21] for the successful movements in this study. Those are defined in the rule book of the International Judo Federation [20] in detail. Therefore, we set N=4. For the first depth, we just pick up the major parts of body that form the throwing techniques. In d=2, we try to divide Jigoro Kano’s classification into body positions for initiating a throwing technique: *head height*, *body contact*, *body position*, and *arm position*. The head height relates to the head positions of Tori and Uke. The body contact relates to the state of contact between Tori and Uke’s bodies. The body position relates to the postures and angles of Tori and Uke. The arm position relates the arm actions of Tori and Uke. In d=3, the factors are further divided into 16 factors. Here, we divide the body position and the arm position into six types of finer granularity: *Natural posture* (basic natural posture, right natural posture, left natural posture) and *Defensive posture* (basic defensive posture, right defensive posture, left defensive posture). We determine whether a posture is defensive or not based on the angle of the upper body. As shown in Figure 3a, we define the *Upper body vector*, which is the one from the midpoint between the greater trochanters to the one between the acromions. The *Floor vector* is the vertical upward vector from the floor. We define the angle of the upper body vector against the floor vector. We say “Natural” when the angle is less than 45 degrees, and “Defensive” when the angle is more than 45 degrees. Regarding the direction of the shoulders and feet, as shown in Figure 3b, we define the *front vector*, which directs the front of the body by applying the vertical bisector of the line between the acromions or ankles. The *position vector* directs the midpoint between the acromions or ankles to the position of the opponent’s umbilicus. We focus on the angle of the position vector against the front vector. We say “Front” when the angle is less than 30 degrees in the left or right side, “Right” when it is greater than 30 degrees in the right side, and “Left” when it is greater than 30 degrees in the left side. Next, regarding the arm position, we focus on arm action and inside–outside relationships of arms. These relate to the technique to move into the subsequent throwing form. These are divided into eight factors regarding left and right arms of Tori and Uke. There are five possible patterns of arm actions: “No effect” when Tori or Uke is not gripping anywhere, “Arm” when Tori or Uke is gripping the opponent’s arm or uniform, “Front” when Tori or Uke is gripping the opponent’s front side of uniform, “Back” when Tori or Uke is gripping the opponent’s back side of the uniform, and “Reverse” when Tori or Uke is gripping some part of opponent’s uniform by crossing the direction (i.e., right/left hand grips the right/left half of the opponent’s uniform). Regarding inside–outside relationship of arms, we say “Inside” when a part of the right/left arm is both inside and below the opponent’s left/right arm, and “Outside” otherwise. Thus, using the candidate factors at d=3 above, we finally drive the correlations for the throwing techniques.

As described above, we define the four categories of judo throwing techniques and 16 candidate factors before initiating them. We extract scenes of the successful throwing techniques from the videos, and mark binary values in the corresponding candidate factors above from the postures before initiating the throw, and create a dataset for the analysis phase.

### 3.3. Data Pickup from Video in Judo Match

**Figure 4:**
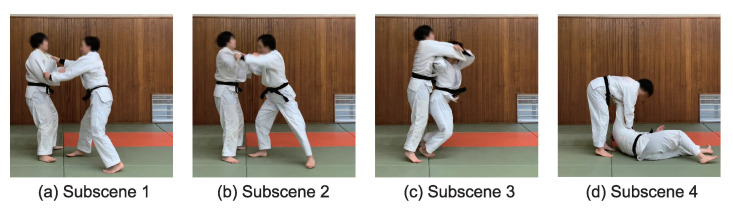
Four subscenes in a scene of Seoi-nage. (**a**) The subscene recognized as the posture right before the throw. (**b**) The subscene when the throw is beginning as the leg of Tori leaves the floor. (**c**) The subscene during the throw. (**d**) The subscene of *Ukemi*.

This section explains how to select the scenes of judo throws and create the dataset from the scenes. We define four subscenes in a throwing scene as shown in Figure 4. In the first subscene, both of Tori’s feet are touching the floor. When Tori begins the throw, one or both feet leave the floor, as in the second subscene. Then, Tori is performing the throw during the third scene. In the final scene, Uke performs *Ukemi* (i.e., the fall breaking, which prevents injury when Uke is thrown by Tori). The posture before initiating a throwing technique is taken from a frame image from the first scene.

In judo, we need to consider the dominant directions of the throwing techniques due to Tori’s dominant direction such as right grip or left grip (i.e., *migi-gumi* or *hidari-gumi*). The rolling directions of a throwing technique differ between the dominant direction. This causes a bias of the collected dataset. In this research, we standardize the directions into the right ones by mirroring the movement of the left ones. When a throw is performed to the right direction, the dataset taken from the video frame image is directly used as the input of the XSM method. On the other hand, in the case when it is performed by the left side, the mirrored subscene image is used as the observation for the input data to the analysis phase.

### 3.4. Statistical Analysis According to $\chi^{2}$ Test and Residual Analysis

In the analysis phase of the XSM method, we apply the $\chi^{2}$ test [29] and the residual analysis [30] to each cross-tabulation of $T(G\left(cf_{j}^{i-1\to i}\right))$ where $1\leq j\leq C^{i-1\to i}$ in d=i are derived from the measurement phase. We call the element in the cross-tabulation the observed frequency $O_{ij}$ where *i* and *j* are the indices of the row and the column, respectively.

We derive the expected frequency $E_{ij}$ regarding $O_{ij}$. It is derived from the following equation where the summations of the frequencies in the *i*-th and the *j*-th columns are defined as $f_{i}$ and $f_{j}$, respectively, and the number of elements in the cross-tabulation is *n*:

$$E_{ij}=\frac{f_{i}\times f_{j}}{n}$$

Furthermore, the $\chi^{2}$ value is derived from the following equation, which is a metric of the deviation between the observed and the expected frequencies:

$$\chi^{2}=\sum_{i=1}^{M}\sum_{j=1}^{N}\frac{\left(O_{ij}-E_{ij}\right)}{E_{ij}}$$

Here, we define the null hypothesis $H_{0}$ and the alternative one $H_{1}$ as “two variables are independent” and “two variables are *not* independent” respectively. The $\chi^{2}$ test performs a hypothesis test under the property that the $\chi^{2}$ values follow the $\chi^{2}$ distribution approximately under an assumption where the null hypothesis $H_{0}$ is correct. When the cross-tabulation is an m×n matrix, the test performs a one-sided test using a $\chi^{2}$ distribution with the degrees of freedom. We apply the significance level of the test to *p*% and examine whether the $\chi^{2}$ value is included in a rejection region of *p*% in the $\chi^{2}$ distribution with the degrees of freedom. The threshold of the rejection region is obtained based on a $\chi^{2}$ distribution table of (m−1)×(n−1) degrees of freedom. If the $\chi^{2}$ value is greater than the threshold, we reject the null hypothesis $H_{0}$ and accept the alternative hypothesis $H_{1}$. During this step, we can see differences among the variables stored in the row and the column of the matrix. Here, we can find combinations of correlated variables between the candidate factors and the successful movements.

The next step examines a correlation between two variables such as a candidate factor and successful movements. We use the residual analysis to numerize how the observed frequencies differ from the expected ones. The residual is derived from the subtraction of those frequencies using the following equations where the standardized residual $e_{ij}$ and its variance $v_{ij}$ of the *i*-th row and the *j*-th column of the cross-tabulation:

$$e_{ij}=\frac{O_{ij}-E_{ij}}{\sqrt{E_{ij}}}$$

$$v_{ij}=\left(1-\frac{f_{i}}{n}\right)\left(1-\frac{f_{j}}{n}\right)$$

The standardized residuals equal the divided values by following their standard deviations, and approximately follow a normal distribution where the mean is zero and the variance is $v_{ij}$. Additionally, we perform a standardized transform using the standardized residuals and the variances. This gives us the adjusted residuals $d_{ij}$ in the row *i* and the column *j* from the following equation:

$$d_{ij}=\frac{e_{ij}}{\sqrt{v_{ij}}}$$

The distribution of the adjusted residuals approximately follows a standard normal distribution where the mean equals to zero and the variance is 1. Thus, we can regard that the value equals to the Z-value in the standard normal distribution.

When $|d_{ij}|$ is greater than or equal to 1.96 in a test for 5% significance level, we can find a correlation between the *i*-th successful movement and the *j*-th candidate factor. We call the candidate factor the *key factor*. We also call the residual value the *key factor threshold*. Here, according to the property of the residual analysis, we can find that the key factor induces the successful movement when $d_{ij}$ is positive, and the factor is regarded as failure when it is negative. However, the XSM method analyzes only the scenes of the successful movements but excludes the failure movements. Therefore, this method does not allow us to evaluate the significance when the adjusted residuals are negative (i.e., $d_{ij}$ < −1.96). Thus, we only consider that the positive cases of the adjusted residuals (i.e., $d_{ij}$ > 1.96) are observed as the key factors.

As explained above, the XSM method is a simple correlation analysis applying $\chi^{2}$ test and residual analysis to derive the key factors and key elements from the successful target movements of the postures before initiating the movements in video scenes. We just define the candidate factors against the successful movements and pick up the factors from the scenes. This allows us to conduct a new coaching or training method for the movements with external influences such as combat sports.

Here, let us discuss the complexity of the XSM method. The main calculation of the XSM method is the $\chi^{2}$ test. The complexity of the analysis phase is equivalent to the calculation of the standardized residual $e_{ij}$ from a cross-tabulation. The total number of the cross-tabulations in a depth *d* is $C^{d\to d+1}$ because each candidate factors has a group of the child factors. Each cross-tabulation has $N\times M(cf_{k}^{i\to i+1})$ elements, where $1\leq k\leq C^{i\to i+1}$. Therefore, the complexity of the standardized residual in a depth is $O(N\cdot M\left(cf_{k}^{i\to i+1}\right))$. When *D* is large enough, assume that the average number of the candidate factors of all $M(cf_{k}^{D-1\to D})$ is $M_{avg}^{D-1\to D}$, the complexity of the XSM method becomes approximately $O(N\cdot M_{avg}^{D-1\to D}\cdot C^{D-1\to D})$.

## 4. Experimental Analysis Examples

### 4.1. Experimental Setup

Now, we show examples of the applications of the XSM method applying to judo throwing techniques and confirm the validity. As a dataset of the scenes that include the throwing techniques, we will use the video database recorded in the 2019 World Judo Championships, the 2018 World Judo Championships, the 2019 World Masters, and the 2018 World Masters available from the IJF website. We collected 781 successful scenes of single throwing techniques except counter and combinational ones. Table 2 shows the statistics of the scenes. As combinations of attribute information for performing the projection step in the XSM method, we can consider many patterns such as weight classes, world ranking of the athletes and so on. In this paper, as examples of the projection, we perform two cases of all throwing techniques without any projection and with a projection by gender difference.

### 4.2. Analysis Example without Projection

Table 3 shows the results of the XSM method analysis for all scenes without any projection regarding four classifications of the throwing techniques. We have not observed any significant characteristic regarding the following three factors: (a) head height, (b) inside–outside relationships of Tori’s left arm and (c) inside–outside relationship of Uke’s right arm. Besides, we have found characteristics regarding the other 13 candidate factors as the key factors.

However, we reject three candidate factors that did not result as key factors. We can utilize the results as the factors that do not induce successful throwing techniques. For example, we expected that the head height influences Tori’s posture related to its center of gravity. However, the result does not show any significant effect of the factor. This means that the factor is excluded reasonably because the head movement does not influence inducing a throwing technique by the weight shift. Additionally, according to [21], the inside–outside relationships of Tori’s left arm and Uke’s right arm do not influence the rotation movement of the throw because the *Hikite* (i.e., pulling hand) just needs to pull Uke’s body. Therefore, we observe that those factors are reasonably excluded from the key ones.

On the other hand, let us discuss the key factors that induce the successful throws referring to the guides for throwing techniques illustrated in [21]. The results in Table 3 show 13 factors in red. We classify those into the following five categories and observe the reasons: (a) body contact, (b) upper bodies of Tori and Uke, (c) positions of Tori and Uke, (d) Tori’s *Tsurite* (i.e., the lifting hand) and (e) Tori’s Hikite.

Category (a) can be explained reasonably because it induces the beginning of throws with affecting to the rotation movements. According to [21], the hand techniques require a large rotation. This movement needs an adequate distance between Tori and Uke. On the other hand, the sacrifice techniques are performed closely between Tori and Uke.

Category (b) relates to the angle of the upper body during the throw. Focusing on the hand and the hip techniques, Tori should raise the upper body before a throw. Regarding the foot techniques, Tori throws Uke to Uke’s backside. This requires Tori to rise the upper body before the throw. Additionally, in the beginning of the sacrifice techniques, both Tori and Uke should keep their upper bodies low.

Regarding (c), four factors have been selected as the key factors: (c-1) Uke’s position against Tori’s shoulders, (c-2) Uke’s position against Tori’s legs, (c-3) Tori’s position against Uke’s shoulders and (c-4) Tori’s position against Uke’s legs. According to [21], during the hand technique, Tori needs to stand on the right side relative to the shoulder and the feet of Uke because Tori performs a large *Kuzushi* (i.e., balance breaking) against Uke in the direction of the Hikite. Moreover, to increase the rotation to the left side of Tori, Tori twists themself by turning their feet toward Uke and by turning Tori’s shoulders toward the left side of Uke. Additionally, it is important for the successful throw in the following three points. First, Tori needs to initiate the hip techniques by facing in front of Uke. Second, Tori needs to initiate a foot technique when Tori’s posture becomes the posture of the right half of the body in a *Kenka-yotsu* (i.e., asymmetrical grips by the two opponents) position. Finally, Tori needs to initiate a sacrifice technique when the posture becomes the left half of the body in the Kenka-yotsu position. Therefore, we confirm that the result reasonably shows the key factors.

Regarding (d), four key factors have been selected: (d-1) Tori’s right arm action, (d-2) inside–outside relationship of Tori’s right arm, (d-3) Tori’s left arm action, and (d-4) inside–outside relationship of Tori’s right arm. The literature [21] explains that the hand techniques require both rotation and movement that Tori dives into the Uke’s chest. Therefore, not to let Uke know the beginning of a hand technique, Tori should hold Uke’s hand suddenly when Uke and Tori are both not offensive. Thus, we confirm that the result reasonably shows the key factors regarding the hand techniques.

Regarding (e), two key factors have been selected: Tori’s left arm action and Uke’s right arm action. These key factors are related to the hand technique. Most of the results are tightly related to *Ippon-seoi-nage*. As mentioned in [21], gripping the fronts of uniforms by both Tori and Uke causes a successful hand technique. Therefore, we observe that those factors are reasonably selected for the key ones.

As explained above, we confirmed that the key factors selected by the XSM method in this example are obviously selected as the causes of the successful throwing technique.

### 4.3. Analysis Example Projected by Gender Difference

Next, let us show another example with a projection in the XSM method regarding the gender difference. During this analysis, we performed two projections based on males and females. The analysis phase of the XSM method processes those datasets, respectively. Table 4 and Table 5 show the results of male and female, respectively. Let us discuss the key factors that differ between genders.

Focusing on the factors, we do not find any significance for the successful throws in the head height as well as the case without considering gender difference discussed in the previous section. Regarding the other factors, we can also find similar characteristics. We do not find any significance regarding the inside–outside relationships of Uke’s right arm as well as the case without gender difference. However, we can find differences in the other factors between genders. Regarding the factors of Uke’s upper body and inside–outside relationships of Tori’s left arm, we confirmed that the key factors differ from the ones in the result without gender difference. Let us discuss these two factors in detail.

Uke’s upper body shows significance only for males. According to the result, we confirmed that the male players only pay attention to the angle of the factor during a throw. The main reason for the result relates to the difference of body core between men and women [31]. The female case shows a large diversity in the way Uke’s balance collapses. It was not selected as a key factor. On the other hand, the inside–outside relationships of Tori’s left arm show a significance only for females. We found that the grip from the outside against the Uke’s right arm works effectively against a sacrifice technique. We can explain the difference by volume and length of muscle between genders [32].

Through the example above, we confirmed the finding of key factors effectively from an analysis of the XSM method based on a specific dataset projected by some attributes.

### 4.4. Discussion for Experimental Analysis

We have evaluated the XSM method using the realistic data captured from the matches of the championships. Especially, focusing on the ability of the method for exploiting the key factors, we used data for the candidate factors acquired from the realistic scenes of the successful movements. According to the examples of analysis, we confirmed that the XSM method finds key factors from candidate factors gathered from the video scenes. Remarkably, the XSM exploits the obvious key factors from data of the matches without any biases that can be caused by an artificial experiment sequence. We have discussed the validity of the key factors by confirming the biomechanical aspects. Thus, the XSM method effectively visualizes the key factors. We also have shown the validity using a dataset with a projection. Even when we apply the projection regarding attributes, we have confirmed that the XSM also clearly exploits and visualizes the key factors.

To utilize the results from our method, it is effective to apply different groups of datasets to the analysis. Then, we can compare the results among the groups. The difference discovered during comparison of the key factors among the groups clarifies the main key factors for the successful throw from the posture before initiating the throw depending on the attributes of the athletes. The conventional biomechanics approach derives the factors from physical models. However, in combat sports, it was hard to find the key factors from the models because the players have factors among them. However, we showed that the XSM method easily derives the key factors by gathering the possible factors observed from the videos.

To introduce the XSM method into the coaching and training, it is effective for coaches to refer to the key factors from the analysis and to observe the related body parts. If we can not find the possible key factors, we can just put all available factors to the XSM method. Then, it will find the body parts that induce the successful movements. Athletes can also utilize this method for self-training. As described above, we can apply the XSM method to factors selected based on existing kinematic knowledge and selected based on the individual characteristics of the target movement. The key factors that lead to the successful movement will help us know some unknown affordance of movement.

## 5. Conclusions

This paper proposed a novel method for finding the factors from postures before initiating a successful movement. We have focused on the throwing techniques in judo. Although the conventional training methods for movement use videos, they do not find the factors that induce the successful movements. Additionally, in combat sports, we focused that there is no method to analyze the factors that lead to the successful movements. Therefore, currently the athletes rely on advice from their coaches. According to the background, we proposed an analyzing system that outputs the key factors for the success, called the XSM method. The system analyzes the factors from the posture before initiating the successful movements, and then, outputs the key factors related to the movements. We have shown examples of the XSM method analysis for judo throws by using the posture information before initiating the successful throws observed from videos. The result showed that the key factors outputted by our method are consistent to the coaching method of judo throwing techniques. We also confirmed another experiment with projection by using attributes such as gender difference. The analysis effectively outputs the appropriate key factors according to the difference of the attributes. Thus, we have proposed a novel system to find factors that lead to successful movements from the posture before initiating the target movement.

For future works, we have two main issues to be addressed. One is to verify the validity of the XSM method when the number of candidate factors is increased because the $\chi^{2}$ test has a disadvantage regarding accuracy as the cross-tabulation becomes large. We will search for a solution by providing a method for making a tree of the candidate factors. It would be effective to apply the XSM method to the more complicated sports without categories of successful movements such as boxing. Another is to implement a training system using the XSM method, which includes a graphical interface to work with videos. Then, we will provide self-training systems for combat sports.

## Figures and Tables

**Table 1:** Candicate factors for each depth regarding the throwing techniques in judo.

Candidate Factors		
**Depth = 1**	**Depth = 2**	**Depth = 3**
Head height	Head height	No difference
		Tori’s chin above Uke’s parietal
		Tori’s parietal below Uke’s chin
Body contact	Body contact	Untouched
		Touched
Body position	Tori’s upper body	Natural
		Defensive
	Uke’s upper body	Natural
		Defensive
	Uke’s position against Tori’s shoulders	Front
		Right
		Left
	Uke’s position against Tori’s legs	Front
		Right
		Left
	Tori’s position against Uke’s shoulders	Front
		Right
		Left
	Tori’s position against Uke’s legs	Front
		Right
		Left
Arm position	Tori’s right arm action	No effect
		Arm
		Front
		Back
		Reverse
	Inside-outside relationship of Tori’s right arm	Outside
		Inside
	Tori’s left arm action	No effect
		Arm
		Front
		Back
		Reverse
	Inside-outside relationship of Tori’s left arm	Outside
		Inside
	Uke’s right arm action	No effect
		Arm
		Front
		Back
		Reverse
	Inside-outside relationship of Uke’s right arm	Outside
		Inside
	Uke’s left arm action	No effect
		Arm
		Front
		Back
		Reverse
	Inside-outside relationship of Uke’s left arm	Outside
		Inside

**Table 2:** The numbers of scenes in the classifications of throwing techniques used for the experiments.

Techniques	Male	Female	Total
Hand	121	73	194
Hip	65	66	131
Foot	169	128	297
Sacrifice	65	94	159

**Table 3:** Results of the XSM method without projection focusing on the throwing techniques in judo.

		Classification of Throwing Techniques			
Key Factor	Action	Hand	Hip	Foot	Sacrifice
Head height	No difference	-	-	-	-
	Tori’s chin above Uke’s parietal	-	-	-	-
	Tori’s parietal below Uke’s chin	-	-	-	-
Body contact	Untouched	**3.03**	1.35	−1.48	−3.13
	Touched	−4.02	−1.56	1.37	**2.50**
Tori’s upper body	Natural	**2.30**	**2.18**	0.73	−6.68
	Defensive	−2.72	−2.69	−0.76	**4.52**
Uke’s upper body	Natural	1.31	0.48	**1.99**	−5.22
	Defensive	−1.43	−0.50	−2.19	**3.81**
Uke’s position against Tori’s shoulders	Front	1.54	**3.11**	−2.24	−2.50
	Right	−5.51	−4.04	**5.71**	−1.04
	Left	**2.76**	−0.90	−14.72	**3.51**
Uke’s position against Tori’s legs	Front	**3.06**	1.89	−5.01	−0.05
	Right	−6.35	−1.76	**6.33**	−2.96
	Left	1.74	−0.84	−6.55	**2.92**
Tori’s position against Uke’s shoulders	Front	−1.61	**2.94**	1.24	−3.07
	Right	**2.72**	−8.79	−4.25	**3.82**
	Left	−1.71	0.76	**1.98**	−2.13
Tori’s position against Uke’s legs	Front	−0.60	**3.24**	−1.66	−0.81
	Right	**2.63**	−7.11	−2.36	**2.67**
	Left	−2.83	−0.12	**3.51**	−2.73
Tori’s right arm action	No effect	**4.82**	−2.62	−4.81	−0.72
	Arm	−4.16	**5.59**	−5.81	1.70
	Front	0.90	−8.10	**2.56**	0.13
	Back	−12.32	1.01	**5.17**	−3.06
	Reverse	**2.61**	−12.34	−1.31	1.04
Inside-outside relationship of Tori’s right arm	Outside	−4.49	**3.09**	1.33	−0.37
	Inside	**3.48**	−4.31	−1.42	0.36
Tori’s left arm action	No effect	0.56	0.27	0.81	−3.14
	Arm	−2.52	**2.05**	**2.08**	−2.28
	Front	**3.20**	−0.78	−1.60	−1.73
	Back	−3.36	−4.43	−2.84	**4.43**
	Reverse	−0.09	−1.20	−0.01	0.85
Inside-outside relationship of Tori’s left arm	Outside	-	-	-	-
	Inside	-	-	-	-
Uke’s right arm action	No effect	0.56	0.36	1.16	−3.06
	Arm	−3.24	1.77	**2.44**	−2.24
	Front	**3.02**	0.18	−5.60	1.64
	Back	−1.45	−4.27	−1.06	**3.48**
	Reverse	−0.81	−2.41	**2.76**	−3.42
Inside-outside relationship of Uke’s right arm	Outside	-	-	-	-
	Inside	-	-	-	-
Uke’s left arm action	No effect	**4.34**	−0.53	−5.80	0.35
	Arm	0.55	−3.41	−0.76	**2.52**
	Front	−5.56	1.61	**3.48**	−2.34
	Back	−3.28	1.86	1.80	−2.11
	Reverse	0.02	−0.23	0.18	−0.04
Inside-outside relationship of Uke’s left arm	Outside	**4.37**	−2.40	−4.03	1.46
	Inside	−6.41	**2.00**	**3.38**	−1.64

**Table 4:** Results of the XSM method with projection by male focusing on the throwing techniques in judo.

		Classification of Throwing Techniques			
Key Factor	Action	Hand	Hip	Foot	Sacrifice
Head height	No difference	-	-	-	-
	Tori’s chin above Uke’s parietal	-	-	-	-
	Tori’s parietal below Uke’s chin	-	-	-	-
Body contact	Untouched	1.78	**2.14**	−1.34	−3.11
	Touched	−2.14	−3.15	1.23	**2.24**
Tori’s upper body	Natural	1.66	1.81	1.18	−7.90
	Defensive	−1.93	−2.35	−1.28	**4.16**
Uke’s upper body	Natural	1.16	0.51	**1.97**	−7.11
	Defensive	−1.28	−0.55	−2.24	**3.88**
Uke’s position against Tori’s shoulders	Front	0.86	1.84	−2.89	0.57
	Right	−5.06	−2.29	**5.00**	−3.44
	Left	**2.69**	−0.60	−10.23	1.83
Uke’s position against Tori’s legs	Front	**2.30**	0.40	−4.83	**2.11**
	Right	−4.87	0.28	**4.97**	−8.12
	Left	1.24	−1.26	−2.90	1.79
Tori’s position against Uke’s shoulders	Front	−0.06	**2.44**	−1.19	−1.09
	Right	1.50	−5.73	−0.94	1.66
	Left	−2.05	−0.42	**2.10**	−0.86
Tori’s position against Uke’s legs	Front	0.58	**2.26**	−2.63	0.07
	Right	1.68	−4.26	−0.10	0.38
	Left	−3.19	−0.48	**2.61**	−0.48
Tori’s right arm action	No effect	**4.02**	−2.19	−5.63	−0.20
	Arm	−3.70	**4.43**	−6.36	**2.59**
	Front	0.26	−6.32	**2.77**	−0.89
	Back	−9.00	0.26	**3.86**	−1.30
	Reverse	**2.39**	-	0.46	−4.84
Inside-outside relationship of Tori’s right arm	Outside	−3.50	**3.22**	−0.01	0.30
	Inside	**2.75**	−5.59	0.01	−0.31
Tori’s left arm action	No effect	−0.57	0.42	1.02	−2.51
	Arm	−0.58	1.55	1.26	−3.16
	Front	1.20	−0.32	−0.77	−0.32
	Back	−1.84	−3.93	−1.74	**3.06**
	Reverse	0.83	-	−1.34	1.33
Inside-outside relationship of Tori’s left arm	Outside	-	-	-	-
	Inside	-	-	-	-
Uke’s right arm action	No effect	0.10	−0.24	0.49	−0.62
	Arm	−2.24	**2.07**	1.32	−2.16
	Front	**2.60**	−1.09	−3.24	0.96
	Back	−1.94	−1.52	−0.10	**2.25**
	Reverse	−0.10	−1.99	1.82	-
Inside-outside relationship of Uke’s right arm	Outside	-	-	-	-
	Inside	-	-	-	-
Uke’s left arm action	No effect	**3.98**	−0.06	−6.38	0.24
	Arm	−0.31	−0.79	−0.15	1.19
	Front	−6.41	0.62	**3.79**	−1.78
	Back	−1.20	0.66	1.02	−1.05
	Reverse	0.48	-	−1.13	1.12
Inside-outside relationship of Uke’s left arm	Outside	**4.23**	−3.72	−2.76	0.84
	Inside	−6.96	**2.59**	**2.35**	−0.94

**Table 5:** Results of the XSM method with projection by female focusing on the throwing techniques in judo.

		Classification of Throwing Techniques			
Key factor	Action	Hand	Hip	Foot	Sacrifice
Head height	No difference	-	-	-	-
	Tori’s chin above Uke’s parietal	-	-	-	-
	Tori’s parietal below Uke’s chin	-	-	-	-
Body contact	Untouched	**2.75**	−0.44	−0.66	−1.68
	Touched	−4.71	0.41	0.63	1.43
Tori’s upper body	Natural	1.58	1.27	−0.24	−-2.67
	Defensive	−1.93	−1.50	0.24	**2.15**
Uke’s upper body	Natural	-	-	-	-
	Defensive	-	-	-	-
Uke’s position against Tori’s shoulders	Front	0.90	**2.67**	−0.53	−3.47
	Right	−2.30	−3.66	**3.18**	0.18
	Left	1.16	−0.71	−10.53	**2.99**
Uke’s position against Tori’s legs	Front	1.59	**2.33**	−2.56	−1.55
	Right	−3.46	−3.28	**4.21**	−0.58
	Left	1.29	−0.10	−7.95	**2.30**
Tori’s position against Uke’s shoulders	Front	−2.89	1.79	**2.78**	−2.90
	Right	**2.55**	−6.92	−5.70	**3.40**
	Left	−0.35	1.32	0.55	−2.13
Tori’s position against Uke’s legs	Front	−1.86	**2.34**	0.29	−1.11
	Right	**2.21**	−5.96	−3.64	**2.89**
	Left	-0.85	0.34	**2.31**	−3.48
Tori’s right arm action	No effect	**2.79**	−1.60	−1.52	-0.91
	Arm	−1.99	**3.40**	−2.06	−0.50
	Front	0.81	−4.93	0.47	1.25
	Back	−8.51	1.10	**3.47**	−3.14
	Reverse	1.32	−6.51	−2.86	**2.27**
Inside-outside relationship of Tori’s right arm	Outside	−2.35	0.82	**2.22**	−1.33
	Inside	1.86	−0.91	−2.73	1.18
Tori’s left arm action	No effect	1.20	−0.07	−0.01	−2.04
	Arm	−3.64	1.37	1.68	−0.40
	Front	**3.31**	−0.79	−1.56	−2.15
	Back	−3.19	−2.69	−2.27	**3.19**
	Reverse	−2.09	0.29	0.98	−0.44
Inside-outside relationship of Tori’s left arm	Outside	−1.41	−0.03	−1.36	**2.54**
	Inside	1.21	0.03	1.23	−3.57
Uke’s right arm action	No effect	0.66	0.75	1.14	−3.93
	Arm	−2.80	0.36	**2.08**	−0.87
	Front	1.81	1.06	−4.68	1.14
	Back	−0.13	−6.51	−1.65	**2.71**
	Reverse	−0.97	−1.63	**2.13**	−1.89
Inside-outside relationship of Uke’s right arm	Outside	-	-	-	-
	Inside	-	-	-	-
Uke’s left arm action	No effect	1.83	−0.66	−1.98	0.47
	Arm	1.22	−4.67	−0.94	**2.24**
	Front	−1.64	1.65	0.77	−1.46
	Back	−4.20	1.84	1.58	−2.13
	Reverse	−0.34	0.52	0.90	−2.51
Inside-outside relationship of Uke’s left arm	Outside	1.70	0.06	−3.01	1.29
	Inside	−2.13	−0.06	**2.46**	−1.47

## Data Availability

Not applicable.

## References

[1] Martens R. (2012). Successful Coaching.

[2] Wulf G. (2007). Attention and Motor Skill Learning.

[3] Richard B., Anne O. (2020). Athlete-centred coaching: Perspectives from the sideline. Sport. Coach. Rev..

[4] Hackman J., Wageman R. (2005). A theory of team coaching. Acad. Manag. Rev..

[5] Hawkins P. (2017). Leadership Team Coaching: Developing Collective Transformational Leadership.

[6] O’Donoghue P. (2006). The use of feedback videos in sport. Int. J. Perform. Anal. Sport.

[7] Yamagiwa S., Ohshima H., Shirakawa K. (2015). Development of Skill Scoring System for Ski and Snowboard. International Congress on Sports Science Research and Technology Support.

[8] Yamagiwa S., Kawahara Y., Tabuchi N., Watanabe Y., Naruo T. Skill grouping method: Mining and clustering skill differences from body movement BigData. Proceedings of the 2015 IEEE International Conference on Big Data (Big Data).

[9] Kurt M., Guenter S. (1995). Bewegungslehre: Sportmotorik.

[10] Wilson B.D. (2008). Development in video technology for coaching. Sports Technol..

[11] Palao J.M., Hastie P.A., Cruz P.G., Ortega E. (2015). The impact of video technology on student performance in physical education. Technol. Pedagog. Educ..

[12] Guadagnoli M., Holcomb W., Davis M. (2002). The efficacy of video feedback for learning the golf swing. J. Sport. Sci..

[13] Emmen H., Wesseling L., Bootsma R., Whiting H., Wieringen P. (1985). The effects of video-modeling and video-feedback on the learning of the tennis serve by novices. J. Sport. Sci..

[14] Marta G., Simona F., Andrea C., Dario B., Stefano S., Federico V., Marco B., Francesco B., Stefano M., Alessandra P. (2020). Wearable Biofeedback Suit to Promote and Monitor Aquatic Exercises: A Feasibility Study. IEEE Trans. Instrum. Meas..

[15] Qiu S., Wang Z., Zhao H., Hu H. (2016). Using Distributed Wearable Sensors to Measure and Evaluate Human Lower Limb Motions. IEEE Trans. Instrum. Meas..

[16] Salim M.S., Lim H.N., Salim M.S.M., Baharuddin M.Y. Motion analysis of arm movement during badminton smash. Proceedings of the 2010 IEEE EMBS Conference on Biomedical Engineering and Sciences (IECBES), IEEE.

[17] Ghasemzadeh H., Loseu V., Guenterberg E., Jafari R. Sport Training Using Body Sensor Networks: A Statistical Approach to Measure Wrist Rotation for Golf Swing. Proceedings of the Fourth International Conference on Body Area Networks.

[18] Loog M., de Ridder D. Local Discriminant Analysis. Proceedings of the 18th International Conference on Pattern Recognition (ICPR’06).

[19] Ball K.A., Best R.J. (2007). Different centre of pressure patterns within the golf stroke II: Group-based analysis. J. Sport. Sci..

[20] Federation I.J. International Judo Federation, Sport and Organization Rules, Version: 8 July 2020. https://www.ijf.org/.

[21] Kano J. (2013). Kodokan Judo: The Essential Guide to Judo by Its Founder Jigoro Kano.

[22] Dopico C.X., Iglesias-Soler E., Carballeira E. (2014). Classification of judo motor skills: Tactical and motor criteria approach. Arch. Budo. Sci. Martial Arts Extrem. Sport..

[23] Mayo X., Dopico C.X., Iglesias-Soler E. (2019). An analysis model for studying the determinants of throwing scoring actions during standing judo. Sports.

[24] Pucsok J., Nelson K., Ng E. (2001). A kinetic and kinematic analysis of the harai-goshi judo technique. Acta Physiol. Hung..

[25] Takanori I., Michiyoshi A., Yuta S., Yasuto K. (2018). Kinematic comparison of the seoi-nage judo technique between elite and college athletes. Sports Biomech..

[26] Rodney I., Benjamin J. (2003). A kinematic analysis of a judo leg sweep: Major outer leg reap—Osoto-gari. Sports Biomech..

[27] Bianca M., Carlos R.H., Ursula F.J., Michel C., Emerson F. (2011). Objectivity of FRAMI-Software for Judo Match Analysis. Int. J. Perform. Anal. Sport.

[28] Giovani M., Emerson F., José R.J., Turibio Leite Barros N. (2010). Structural Analysis of Action and Time in Sports: Judo. J. Quant. Anal. Sport..

[29] Harald C. (2016). Mathematical Methods of Statistics (PMS-9).

[30] Shelby J. (1973). The Analysis of Residuals in Cross-Classified Tables. Biometrics.

[31] Marras W., Jorgensen M., Granata K., Wiand B. (2001). Female and male trunk geometry: Size and prediction of the spine loading trunk muscles derived from MRI. Clin. Biomech..

[32] Miller A.E.J., MacDougall J.D., Tarnopolsky M.A., Sale D.G. (1993). Gender differences in strength and muscle fiber characteristics. Eur. J. Appl. Physiol. Occup. Physiol..

